# Characterization of tissue heterogeneity by contrast-enhanced cardiovascular magnetic resonance imaging is a powerful predictor of ventricular tachyarrhythmias on ambulatory holter ECG in hypertrophic cardiomyopathy

**DOI:** 10.1186/1532-429X-11-S1-O20

**Published:** 2009-01-28

**Authors:** Caitlin J Harrigan, A Selcuk Adabag, Evan Appelbaum, Kevin S Heffernan, John R Lesser, James E Udelson, Warren J Manning, Barry J Maron, Martin S Maron

**Affiliations:** 1 Tufts Medical Center, Boston, MA USA; 2 Minneapolis Heart Institute Foundation, Minneapolis, MN USA; 3 Perfuse Core Lab, Boston, MA USA; 4 Beth Isreal Deaconess Medical Center, Boston, MA USA

**Keywords:** Cardiovascular Magnetic Resonance, Standard Deviation, Late Gadolinium Enhancement, Hypertrophic Cardiomyopathy, Ventricular Tachyarrhythmia

## Background

Cardiovascular magnetic resonance with late gadolinium enhancement (LGE-CMR) identifies areas of myocardial scarring in patients with hypertrophic cardiomypathy (HCM). The presence of LGE identifies HCM patients at risk for ventricular tachyarrhythmias, which is an independent predictor of sudden death in this disease. In this study, we sought to determine whether regions of abnormal tissue at the confluence of both viable myocardium and fibrosis ("border zone") (BZ) are more predictive of ventricular tachyarrhythmias than areas of myocardial scarring alone.

## Methods

Cine CMR and LGE-CMR were performed in 145 HCM patients (42 ± 15 years; 74% male) from two HCM referral centers using standard techniques (0.2 mmol/kg gadolinium-DTPA). LGE was determined by a blinded, independent reader using grayscale thresholds of both 4 and 6 standard deviations (SD) above the mean of normal, remote myocardium. BZ was determined as the difference between the 4 SD and 6 SD thresholds. All subjects underwent 24-h ambulatory Holter electrocardiogram (ECG) within 7.8 ± 8.3 weeks of the CMR

## Results

The amount of myocardial scarring (6 SD) alone was 5.5 ± 7.2% of the LV myocardium, while the amount of BZ was 6.5 ± 4.6% of the LV myocardium. Runs of non-sustained ventricular tachycardias (NSVT) were present in 23 patients (16%), ventricular couplets were present 39 patients (27%), while premature ventricular couplets (PVCs) were present in 119 patients (82%). There was no significant association with the extent of myocardial scarring and the occurrence of NSVTs, couplets, and PVCs (overall p = 0.05). However the extent of BZ was significantly associated with the occurrence of NSVTs, couplets and PVCs (overall p < 0.001).

## Conclusion

These data demonstrate that in HCM patients, the extent of BZ may be a superior predictor of ventricular tachyarrhythmias than myocardial scarring alone (using a grayscale thresholding technique of 6 SD above normal myocardium). This study indicates the need for follow-up studies evaluating the independent prognostic value of BZ in risk stratification strategies in HCM patients.

